# Antidiabetic and antioxidant potential of *Gardenia latifolia* in type-2 diabetic rats fed with high-fat diet plus low-dose streptozotocin

**DOI:** 10.15537/smj.2022.43.8.20220258

**Published:** 2022-08

**Authors:** Ali M. Alshabi, Ibrahim A. Shaikh

**Affiliations:** *From the Department of Clinical Pharmacy (Alshabi) and from the Department of Pharmacology (Shaikh), College of Pharmacy, Najran University, Najran, Kingdom of Saudi Arabia.*

**Keywords:** *Gardenia latifolia*, high fat diet, type-2 diabetes mellitus, insulin resistance, Saudi Arabia

## Abstract

**Objectives:**

To test the antidiabetic potential of *Gardenia latifolia* extract (GLE) in rats with type 2 diabetes mellitus (T2DM) induced by a high-fat diet (HFD) + streptozotocin (STZ).

**Methods:**

The study was carried out in June 2021. *Gardenia latifolia* powdered leaves were subjected to Soxhlet extraction using ethanol. Male rats were administered a low dose-40 mg/kg STZ by intraperitoneal route following 2 weeks of HFD to induce type-2 diabetic rats (T2DR). Rats were randomized into 5 groups (n=6). Group 1 (normal control; 10 ml/kg normal saline); Group 2 (diabetic control: DC); Group 3 (standard; DR + metformin, 100 mg/kg per oral); Group 4 (DR + GLE 250 mg/kg); Group 5 (DR + GLE 500 mg/kg). The treatment period extended for 2 weeks. Body weight and fasting blood glucose were determined on days 0, 7, and 14. Fasting serum insulin (FSI) levels, fasting blood glucose (FBG), HOMA-IR, antioxidant enzyme level, Insulin tolerance test (ITT), and intraperitoneal glucose tolerance test (IPGTT) tests were estimated.

**Results:**

*Gardenia latifolia* extract exhibited a marked decrease (*p*<0.001) in fasting blood glucose levels. T2DR receiving a higher dose of GLE showed a greater improvement in metabolic indices (FSI, FBG, Homeostatic Model Assessment of insulin resistance). The ITT and IPGTT results demonstrated that GLE could significantly enhance insulin tolerance, glucose tolerance, and antioxidant enzyme levels in T2DR.

**Conclusion:**

*Gardenia latifolia* can be an ideal medicinal plant candidate for treating T2DM, and it should be investigated further for its therapeutic potential.

**D**iabetes mellitus (DM) is a chronic endocrine disorder characterized by improper glucose, lipid, and protein metabolism. Diabetes is defined by a decrease in the circulating levels of insulin (insulin deficit) and a decline in the responsiveness of tissues to insulin (insulin insensitivity).^1^ The disease has spread to epidemic proportions, posing a global threat. Diabetes rates have risen dramatically in Middle Eastern countries over the last 2 decades. Impaired glucose tolerance (IGT) affects 48 million adults in the International Diabetes Federation (IDF) Middle East/North African (MENA) region, predisposing them type 2 diabetes mellitus (T2DM). This figure is predicted to grow to 36 million by 2030, and by 2045, it will be 81 million. In the IDF MENA region in 2021, diabetes was responsible for 796,000 deaths. Saudi Arabia ranks fourth among MENA countries regarding the diabetic population. According to the IDF, around one-fourth of Saudi adults will develop diabetes by 2045.^2^ Type 2 diabetes affects 95% of those who have the disease. The currently available treatments for diabetes are associated with several side effects. As a result, finding alternative remedies and novel preventative measures for type 2 diabetes is crucial. Some of the potential side effects of conventional antidiabetic drugs include hypoglycemia, gastrointestinal tract disturbance, weight gain, skin rash or itching (sulfonylureas); kidney complications, stomach upset, lethargy, metallic taste (biguanides/metformin); flatulence, bloating and diarrhea (alpha-glucosidase inhibitors); liver disease, weight gain, anemia, arthritis (thiazolidinediones); obesity, hypoglycemia (meglitinides); lipodystrophy, hypoglycemia, dizziness, confusion, mood changes, tingling sensations, headaches (insulin-s.c [subcutaneous injections]).^3^

Recent studies show that rats who eat a high-fat diet (HFD) develop insulin resistance but not pre-diabetes or hyperglycemia.^4,5^ It is assumed that the HFD is a better way to begin insulin resistance, which is one of the most early manifestations of T2DM. Streptozotocin is used to cause DM type 1 and 2 by causing beta cell death through DNA alkylation.^6^ High doses of STZ cause severe insulin secretion problems, like type 1 diabetes. However, low doses of STZ have been shown to cause slight insulin secretion problems, as what happens in the later stages of type 2 diabetes.^7,8^ In the current study we used the HFD-low dose STZ model to produce features similar to type 2 diabetes in rats.

Nutraceuticals, which give beneficial health effects and alternatives to contemporary medicine, have attained popularity. They are made up of dietary supplements, herbals, and nutrients, which render them beneficial in promoting health, resisting disease, and enhancing the quality of life. Diabetes mellitus is one of these diseases.^9^ Owing to the presence of therapeutically active phytochemicals, herbal drugs have great potential in treating various disorders. Diabetes is a significant metabolic illness that can be treated with various commercially available drugs. These conventional medications are costly and come with a slew of side effects. Herbal medicines have become increasingly popular in developed countries during the last 2 decades since they are less expensive and have better therapeutic results with fewer adverse effects.^10^

*Gardenia latifolia* is a plant with many medicinal properties. *Gardenia latifolia* is a short deciduous tree with a slender trunk, native to India and Bangladesh. Fruits are used to treat mammary gland affections, fever, and colic. Snake bites, hand and foot sores, and wounds are treated using fruit extract. Caries is treated by applying crushed stem bark that has been cooked in water to the afflicted areas. The bark is used to treat skin disorders. Saponins from bark decrease histamine production and could be used to manage bronchial asthma. The stem bark contains hederagenin, sitosterol and episiaresinolic, siaresinolic, spinosic, and oleanolic acid.^11,12^ Previous studies have reported anti-inflammatory, antimicrobial, antioxidant and invitro-antidiabetic activities of *G. latifolia*.^12,13^ However, the antidiabetic activity of *G. latifolia* in T2DM has not yet been reported. As a result, testing the pharmacological potential of *G. latifolia* in the management of T2DM is highly relevant. The current investigation was performed to investigate the antidiabetic potential of 2 doses of test drug (orally) in T2DM rats produced by high fat dietary modification and low dosage streptozotocin as part of our attempts to establish the therapeutic effects of *G. latifolia*.

## Methods

This preclinical investigation was carried out in June 2021 at Najran University’s College of Pharmacy, Saudi Arabia.

### Collection and extraction of G. latifolia aerial leaves

The ethanolic extract of *G. latifolia* extract (GLE) was used in this study. The leaves were collected from Karnataka University Campus, Dharwad, India. It was identified by Dr. Rajesh Shastri from the Pharmacognosy Department, Faculty of Pharmacy, Soniya Education Trust, Dharwad, Karnataka, India, and a specimen were collected and stored in a herbarium. The leaves were washed, dried in the shade, crushed, and extracted with 500 ml of ethanol at a temperature of 50 degrees (°) Celsius for 12 hours. A Soxhlet apparatus was used (Wollen lab, China). The extract was first dried using a rotary evaporator, lyophilized into powder and then stored for later use.

### Liquid chromatography–mass spectrometry (LC-MS) and fourier transform infrared (FTIR) profiling

The plant extract was analyzed using the LC-MS-8040 (Shimadzu) model. Mass spectrometry was linked to HPLC. The C18 column with 80:20 (methanol/water, v/v) mobile phase ratio. The mobile phase flow rate was 0.2 ml/minutes. Of the sample, 3 µl was analyzed using the electron spray ionization probe’s detection mode. In Mass Spectroscopy, M+1, and M-1 electrospray ionization probe ionization variations were utilized. Fourier transform infrared analysis was also performed as per standard protocol.^12^

The local animal supplier in Najran, Saudi Arabia provided the Wistar rats (male), weighing between 150 to 200 grams. In the normal cages, the animals were kept in groups of 6 rats each. Both fresh water for drinking and feed were offered on an unrestricted basis. The rats were maintained on a cycle of light and dark that lasted for 12 hours. All of the procedures that required the use of laboratory animals were carried out in accordance with the National Institutes of Health Guiding Principles in the care and use of animals.

Streptozotocin and metformin hydrochloride were procured from Merck Sigma Aldrich Co, Darmstadt, Germany. All the other chemicals and reagents used were of analytic quality.

The acute toxicity investigation followed the Organisation for Economic Cooperation and Development-423 guidelines. The animals fasted for a night before being dosed.^15^ An oral feeding catheter was used to provide GLE doses starting from 5 mg/kg body weight (b.w.) to 5000 mg/kg b.w. For the first 24 hours, each animal was closely monitored, with specific attention given to the first 4 hours, and then every day for the following 14 days. Seizures, hyperactivity, sedation, hypothermia, grooming, convulsions, and death were all symptoms of toxicity. Based on the results of the toxicity study, the current research’s test doses were selected.

Najran University’s Scientific Ethical Committee authorized the project, and a certificate of ethics approval was issued with the reference number: 443-41-29343-DS. All studies were carried out in accordance with accepted standards for the ethical treatment of animals around the globe (National Institutes of Health Publications No. 8023, revised 1978).

After a week of acclimatization, all of the animals were classified into 5 groups of equal body weight (n=6) as follows: i) Group 1 (normal control; 10 ml/kg normal saline); ii) Group 2 (diabetic control [DC]); iii) Group 3 (standard drug [DR] + metformin, 100 mg/kg per oral [p.o]); iv) Group 4 (DR + GLE 250 mg/kg p.o); and v) Group 5 (DR + GLE 500 mg/kg p.o).

For 2 weeks, the animals in Group 1 were fed a standard normal pellet diet. The animals in groups 2 to 5 were fed a HFD (66.5% standard pellet feed, 13.5% lard, and 20% sugar) for 2 weeks before receiving 40 mg/kg STZ injected intraperitoneally (i.p.) in 0.5% normal saline to induce type-2 diabetes. The animals were given a 5% glucose solution to avoid initial hypoglycemia for the first 12 hours. The HFD was prepared following a previously described procedure.^16^ Animals’ fasting blood glucose (FBG) was measured 3 days after STZ injection using a digital glucometer with test strips (Accu-Chek Aviva blood glucose meter, Roche Diagnostics, Switzerland), and animals were considered diabetic if their FBG level was >250 mg/dL. The normal control and diabetic control groups were given 10 ml/kg of normal saline orally for 2 weeks. The other groups were given their respective drug treatments daily for 2 weeks as per the experimental design. Every day, the experimental animals were given a freshly prepared suspension of metformin and *G. latifolia* extract in 0.5% gum tragacanth.

All animals’ body weights were measured at the beginning (day 0), the end of week one, and week 2. Similarly, blood samples were taken from overnight fasted rats on days 0, 7, and 14 by tail clipping to determine FBG levels. On day 14, we used a radioimmunoassay method to determine fasting serum insulin using an insulin assay kit (Cisbio International, France). The following formula was used to calculate insulin resistance homeostatic model assessment of insulin resistance (HOMA-IR):^17^

HOMA IR=serum insulin (mmol/L)*(blood glucose (mmol/L)/22.5.

Intraperitoneal glucose tolerance test (IPGTT) was performed on overnight fasted animals at the end of the intervention (day 14) by administering glucose (2 g/kg, i.p.) and determining the blood glucose (BG) levels at 0, 30, 60, and 120-minute intervals. To assess insulin tolerance (ITT), overnight fasted rats were challenged with insulin (2 U/kg, i.v.) 48 hours after the IPGTT. After administering insulin, blood samples were drawn at 0, 10, 20, and 30 minute intervals to evaluate BG levels. To demonstrate the significant change in tolerance, the area under the curve (AUC) was measured utilizing the trapezoid rule.^18^

The 12 hours fasted animals were injected intraperitoneally with ketamine hydrochloride (90mg/kg) and xylene hydrochloride (10mg/kg) to induce anaesthesia on day 16 of the study. Anesthetized rats were sacrificed ethically, and the pancreas was skillfully collected and placed in an ice-cold buffer in Petri dishes. For biochemical estimations, small pieces of tissue were frozen at -80°C in liquid nitrogen. Catalase (CAT), glutathione (GSH), and superoxide dismutase (SOD) levels were determined using homogenized pancreas tissue. The invivo antioxidant activity of CAT was determined using previously described standard procedures.^19-21^

### Statistical analysis

The data were presented with the mean and standard error of the mean. A one-way analysis of variance (ANOVA) was performed to assess the statistical differences, and then the post-Tukeys test was carried out to compare means. All comparisons were considered statistically significant if *p*-value of <0.05. The Graph-Pad Prism version 5 program was deployed for all of the statistical analyses.

## Results

### Acute toxicity study

The extract was tolerated by the rats up to a dose of 5000 mg/kg. They all passed the test with no visible physical and behavioral changes. Hence, we selected 1/10th portion (500 mg/kg p.o.) as the higher dose, and 1/20th part (250 mg/kg p.o.) as the lower dose.

### Fourier transform infrared and LC-MS profiling

**Figure 1:**
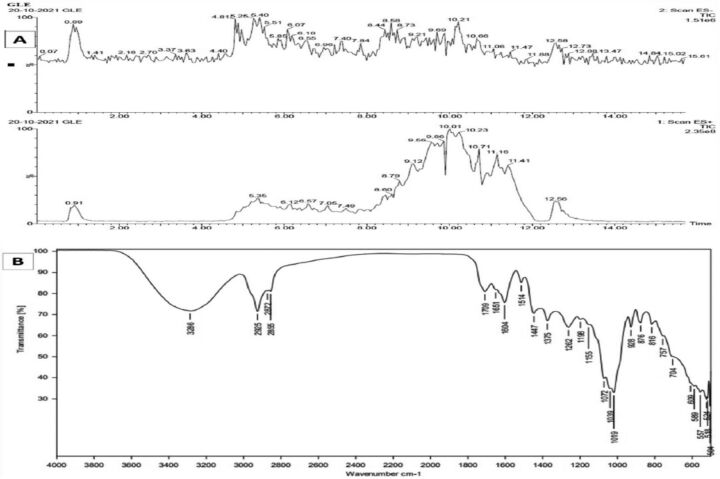
- Spectral analysis of *Gardenia latifolia* (*G. latifolia).* **A**) Liquid chromatography–mass spectrometry of *G. latifolia*; and **B**) fourier transform infrared chromatogram spectra of *G. latifolia*.

The presence of 18 bioactive chemicals was established by searching the National Institute of Standards and Technology [NIST]/ Environmental Protection Agency/ National Institutes of Health Mass Spectral Library (NIST 17) (Figure 1A). Some of the important compounds confirmed by LC-MS include xanthurenic acid; 2-oxo-8-phenylacetyl-1(10),3-guaiadien-12,6-olide; Hetisine; kinetin riboside; 3-(4-Methyl-thiazol-2-yl)-7-(3,4,5-trihydroxy-6-hydroxymethyl-tetrahydro-pyran-2-yloxy)-chromen-4-one; 8-(3,4-Dimethoxy-phenyl)-2,3,10,11-tetramethoxy-5,6,13a-tridehydro-berbinium; 5-[2-(6-hydroxy-4,7-dimethoxy-benzofuran-5-yl)-2-oxo-ethyl]-4-methoxy-6,6-dimethyl-5,6,7,8-tetrahydro-[1,3]dioxolo[4,5-g] isoquinolin-6-ium; 1-(4-Oxo-4H-chromen-3-yl)-2,3,4,9-tetrahydro-1H-beta-carbolin-3-ylcarboxylic acid; N-methyl-norcotamine; belladonnine; Reserpinine; 2-methoxy-phenylacryloyl-lupinine; 6-methyl-5,7a,13,13b-tetraaza-pentaphene-8,14-dione.

The FTIR spectrum shows various peaks in the range of 3286 cm−1 to 504 cm−1, indicating the presence of different functional groups, including C-H stretching, O-H stretching, N-O stretching, C=O stretching, S=O stretching, C=C bending, O-H bending, C-H bending, C-F stretching, representing important bioactive componds belonging to alkane, alkene, alcohol, conjugated aldehyde, esters, sulfoxide, fluorine, halocarbons chemical nature groups (Figure 1B).

### Impact of GLE administration on body weight of rats

**Figure 2:**
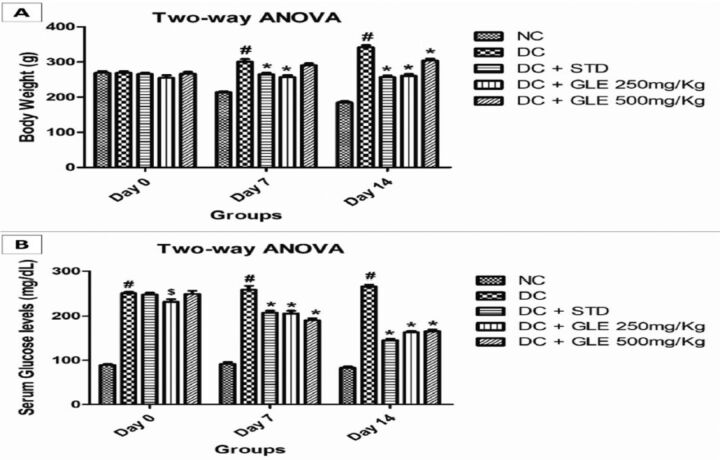
- Impact of *Gardenia latifolia* extract (*GLE)* administation on **A**) body weight of rats; and **B**) glycemic control in HFD-STZ-induced diabetic rats (chronic-dose-14-day treatment). ANOVA: analysis of variance, DC: diabetic control, STD: standard, NC: normal control

Compared to normal rats, diabetic rats had a significant (*p*<0.001) enhancement in body weight at weekly intervals. The GLE (250 mg/Kg) treatment of diabetic rats did not result in a significant rise in the animals’ weight. The weight gain in diabetic rats treated with GLE (500 mg/Kg) was significant on day 7 (*p*<0.05) and day 14 (*p*<0.01), but considerably smaller than in diabetic control rats (*p*<0.001). However, with the standard drug metformin, a significant difference was not observed. However, 2-way ANOVA revealed significant differences in body weight between the groups on days 7 and 14 (Figure 2A).

#### Effect of GLE on glycemic control

*G. latifolia* extract administration significantly lowered FBG levels in diabetic rats compared to baseline values on day 0 (Table 1). Higher doses of GLE were shown to be as effective as the standard drug metformin. The test drug caused a significant decrease in FBG levels commencing on day 7 and lasting until day 14 (*p*<0.01). *Gardenia latifolia* extract showed effective glucose control, similar to metformin, during the 14-day chronic-dose administration. There were no significant variations in glycemic control between the normal control and negative control animals. On days 7 and 14, the results of a 2-way ANOVA showed that there were significant group differences detected within the treatment group compared to diabetic control (inter-group comparisons) (Figure 2B).

**Table 1:** - Effect of *Gardenia latifolia* extract (*GLE)* on fasting bood glucose levels.

Treatment group	Serum glucose levels (mg/dL) (n=6)		
	Day 0	Day 7	Day 14
Normal control	89.0 ± 3.63	91.83 ± 4.86 (-3.3)	82.83 ± 3.79 (6.5)
Diabetic control	251.83 ± 3.87	259.83± 10.79 (-3.6)	267.00 ± 5.77 (-6.2)
Diabetic + metformin (STD)	248.83 ± 5.07	207.83 ± 5.42^†^ (16.4)	145.17 ± 4.59^†^ (41.7)
Diabetic + GLE 250 mg/kg, p.o	232.33 ± 7.41	205.83 ± 7.82* (11.4)	163.67 ± 3.06^†^ (29.4)
Diabetic + GLE 500 mg/kg, p.o	250.33 ± 8.52	190.50 ± 5.27^†^ (23.6)	165.67 ± 4.78^†^ (33.7)

Values are expressed as mean ± standard of mean. The values of reduction in glycemia are shown as percentage (%).

* *p*<0.05,

^†^ *p*<00.01 compared to day zero values of the same group. p.o: per oral, STD: standard

### Effect of GLE on metabolic parameters

In diabetic control rats, we found a significant rise in fasting serum insulin, FBG, and HOMA-IR, compared to normal control rats, demonstrating insulin resistance and glucose intolerance. The high FBG, fasting serum insulin (FSI), and HOMA-IR levels were dramatically reduced after treatment with GLE, indicating a reduction in insulin resistance and improved glycemic management. Compared to the diabetic control group, rats receiving a higher dose of GLE showed a more remarkable improvement in metabolic indices (Table 2).

**Table 2:** - Effect of *Gardinia latifolia* extract (*GLE)* on metabolic indices in high fat diet-streptozotocin diabetic rats

Groups	Fasting serum insulin mmol/L	Fasting blood glucose mmol/L	HOMA-IR
Normal control	10.33 ± 0.88	4.20 ± 0.16	1.94 ± 0.22
Diabetic control	26.67 ± 0.88 *	9.81 ± 0.30*	11.65 ± 0.66*
Diabetic + Metformin (STD)	17.00 ± 1.15***	5.61 ± 0.25^‡^	4.24 ± 0.26^‡^
Diabetic + GLE 250mg/kg p.o	22.33 ± 0.67	8.09 ± 0.26^†^	8.03 ± 0.37
Diabetic + GLE 500 mg/kg p.o	18.67 ± 0.88 ^†^	5.70 ± 0.21^‡^	4.74 ± 0.32^‡^

Each value indicates mean ± standard of mean. One-way analysis of variance followed by post-hoc Tukeys test for multiple comparisions.

* *p*<0.001 compared to normal control group;

† *p*<0.01,

‡ *p*<0.001 compared to diabetic control group. HOMA-IR: homeostatic model assessment of insulin resistance, p.o: per oral

### Impact of GLE intervention on IPGTT and ITT in rats

**Figure 3:**
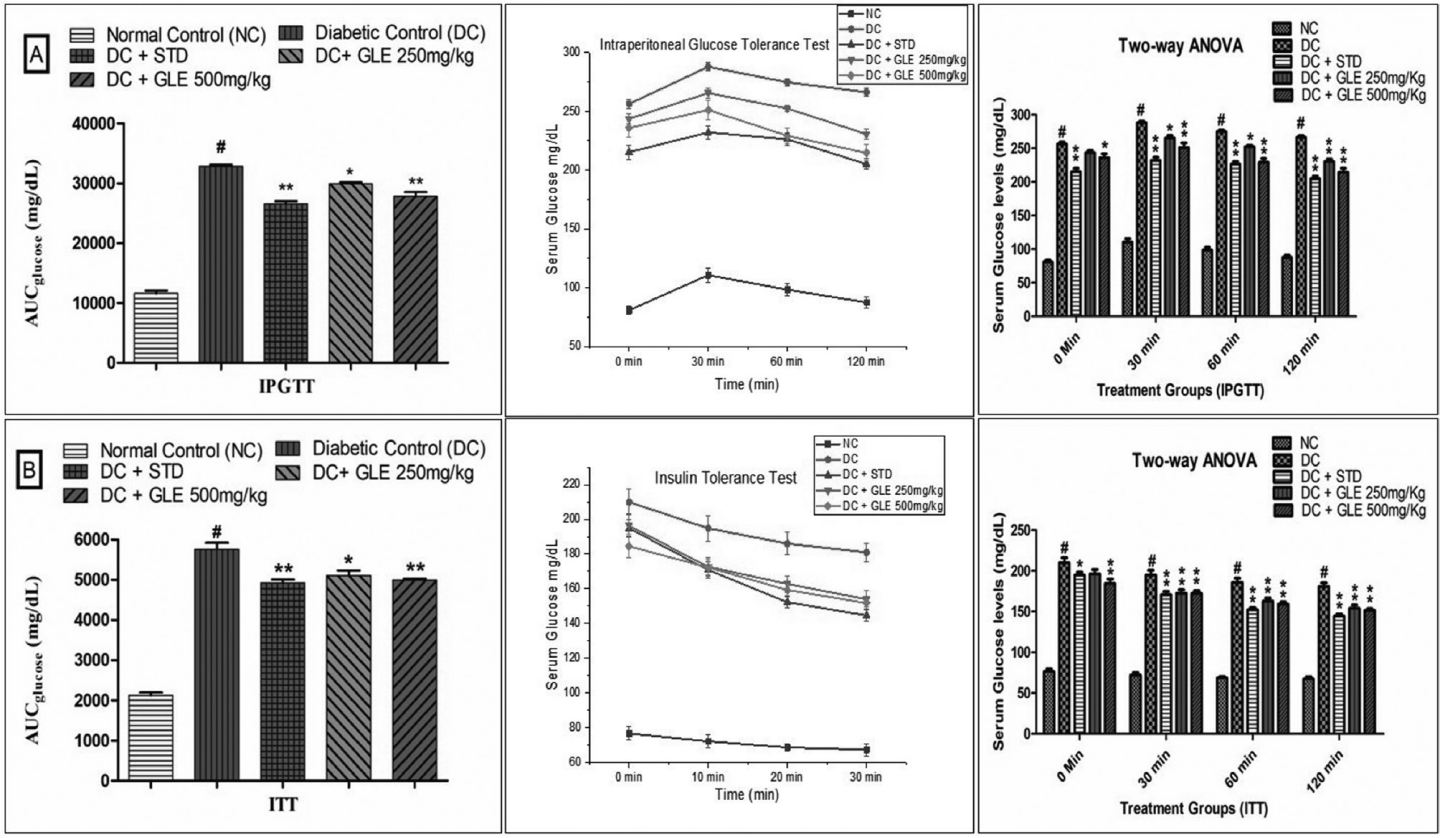
- Effect of *Gardenia latifolia* extract (*GLE)* on **A**) intraperitoneal glucose tolerance test and **B**) insulin tolerance (ITT) in high fat-streptozotocin diabetic rats. ANOVA: analysis of variance, AUC: area of curve, NC: normal control, STD: standard

At the end of day-14 following GLE intervention, IPGTT and ITT were performed to assess glucose and insulin tolerance. After receiving glucose (2g/kg, i.p.), normal control rats’ BG levels did not change significantly; hence, no major changes in AUC were observed. Diabetic rats, on the other hand, showed significantly lower glucose tolerance than normal control rats, as evidenced by higher BG levels and a greater area under the glucose curve. *Garedenia latifolia* extract treatment reduced BG levels and AUC in high HFD-STZ diabetic rats, indicating that the test drug has the potential to improve glucose tolerance (Figure 3A). The insulin tolerance test (ITT) was used to determine the degree of peripheral glucose utilisation. *Gardenia latifolia* extract treatment improved insulin tolerance in the treatment group, as evidenced by lower plasma glucose levels and AUC when compared to the HFD-STZ group (Figure 3B).

### Impact of GLE on the antioxidant levels in-vivo

High fat diet-streptozotocin diabetic rats had considerably lower levels of the endogenous antioxidant enzymes SOD, CAT, and GSH than control rats. Treatment with GLE considerably increased the levels of antioxidant enzymes, indicating the test drugs considerable antioxidant potential (Table 3).

**Table 3:** - Impact of *Gardenia latifolia* extract (*GLE)* on the endogenous invivo antioxidant levels in high fat diet-streptozotocin diabetic rats.

Treatment group	Superoxide dismutase	Catalase	Glutathione
Normal control	9.75± 0.54	15.14 ± 0.37	19.22 ± 0.59
Diabetic control	3.47 ± 0.33*	4.06 ± 0.37*	7.78 ± 0.47*
Diabetic + Metformin	8.42 ± 0.27^†^	10.60 ± 0.40^†^	15.75 ± 0.48^†^
Diabetic + GLE 250mg/kg p.o	6.85 ± 0.24^†^	8.34 ± 0.47^†^	11.96 ± 0.77^†^
Diabetic + GLE 500mg/kg p.o	7.52 ± 0.29^†^	9.88 ± 0.22^†^	13.88 ± 0.48^†^

Data are indicated as mean ± SD (n=6) at *p*<0.05. One-way analysis of variance thereafter by Tukey’s t-test is performed to compare means.

* *p*<0.001, compared to normal control group;

† *p*<0.001 compared to diabetic control group.

## Discussion

As the most common metabolic illness worldwide, diabetes alters the insulin, glucose, cortisol and lipoprotein profiles of the body significantly. Diabetes is widely believed to be on its way to becoming the world’s leading cause of chronic disease. Despite the abundance of research data on diabetes, it is still largely an unsolved medical issue.^22^ In order to avoid and treat this disease, it is critical to follow a healthy diet, get regular exercise, and take nutritional supplements.

Obesity caused by a HFD has been linked to the progression of T2DM.^22^ As a consequence of this, we utilized an animal model that is capable of simulating human T2DM. We used high-fat feeding and streptozotocin to induce T2DM in Wistar rats. The effect of HFD is solely determined by 2 factors: the period of HFD feeding and the HFD composition. Because of the shorter duration of the high-fat diet (2 to 4 weeks), insulin resistance and glucose intolerance may develop. A state of insulin resistance, glucose intolerance, and increased body fat mass can result from consuming a HFD for an extended period of time (at least 5 weeks or more). After the development of insulin resistance, STZ was administered to induce beta-cell dysfunction in the rats. It is a good model for T2DM rats because of the order of pathological events such as insulin resistance, obesity, and β-cell failure.^23^

The leading cause of T2DM is progressive β-cell dysfunction. The discovery of current medications has opened up a new avenue for treating T2DM, although it has to be seen if these drugs can stop the progression of β-cell dysfunction.^24^ Type 2 DM can be treated with various regimens, but some have severe side effects that lead to many other issues.^25^ As a result, more effective treatment regimens with less or no side effects are urgently needed.

Type 2 DM can be prevented by eating functional foods, including fruits, vegetables, and plant-derived products, which reduce insulin resistance and improve homeostasis and insulin profile.^26-28^ *Gardenia latifolia* is a promising plant that has been shown to have a variety of health benefits. As a result, the objective of this study was to analyze the efficacy of *G. latifolia* treatment on T2DM diabetic rats through modulating insulin resistance and oxidative stress.

Insulin resistance causes the body to create more insulin, resulting in increased hunger, elevated blood pressure, and weight gain.^29^ The results of this study show that the T2DM rats had a significant rise in the body weight, suggesting insulin resistance, which was further supported by the elevated HOMA-IR values. *Gardenia latifolia* extract intervention helped optimize the body weight of diabetic rats, with no noticeable gain in the body weight of animals treated with a lower dose (250 mg/Kg). However, administering diabetic rats with GLE (500 mg/Kg) had a considerable gain in the body weight, which lasted into the second week. Similar findings were reported in an earlier study.^30^ In this investigation, rats fed with a HFD showed a rise in body weight, insulin levels, and insulin resistance. The main reason for the rise in insulin levels in the control group could be the pancreas secreting and releasing more insulin to cope with rising glucose levels due to insulin resistance that developed concurrently with obesity.^31,32^

*Gardenia latifolia* extract’s anti-hyperglycemic activity was tested in diabetic rats for 2 weeks. When compared to baseline values (day 0), chronic treatment with the test drug resulted in significant reductions in BG levels on days 7 and 14. On day 7, diabetic rats receiving a higher dose of GLE had considerable BG reductions, with much more significant percentage reductions on day 14. When compared to diabetic rats, GLE showed significant percentage reductions in BG levels, with a 23.6% on day 7 and 33.6% on day 14, which were statistically significant. These findings are in conciliation with the findings of previous studies.^33,34^ Thus, chronic administration of GLE resulted in considerable anti-hyperglycemic benefits, which could be attributable to improved glycemic control due to decreased insulin resistance.

The glucose tolerance test is a basic test used to screen T2DM. In this study, IPGTT was employed to measure glucose tolerance in rats following GLE intervention. Our findings showed that diabetic rats had a significant increase in glucose at 30 minutes, peaked at 60 minutes, and remained stable in the 90 and 120-minute range. The rats receiving GLE, on the other hand, showed a modest increase in glucose levels at 60 minutes, which then dropped at 90 and 120 minutes, which was comparable to the standard drug metformin. Moreover, our findings are consistent with those of Li et al,^35^ who discovered that Schisandra Chinensis modulates glycemic control in HFD/STZ-induced T2DM rats. Similarly, Du et al,^36^ reported the use of Konjac glucomannan in glycemic control in T2DM rats.ssess insulin tolerance following GLE treatment. In HFD-STZ diabetic rats, GLE enhanced insulin tolerance, as evidenced by significant decrease in plasma glucose levels and the area under the ITT curve in the GLE treated group compared to the untreated group. Thus, this study’s ITT and IPGTT results demonstrated that GLE could significantly enhance insulin tolerance and glucose tolerance in HFD fed insulin-resistant rats. These findings are in line with those of Liu et al,^18^ who studied the effects of vaspin on insulin resistance in rats.

In the current study, the diabetic control rats exhibited a significant rise in FBG and FSI, indicating insulin resistance and glucose intolerance. After treatment with GLE, the high FBG and FSI levels were reduced significantly, indicating a reduction in insulin resistance, enhanced peripheral utilization of glucose and better glycemic control. Compared to the diabetic control group, rats given a larger dose of GLE improved their metabolic indicators substantially. Previous investigations by Liu et al^18^ and Hazman et a,^30^ using crocin and vaspin showed similar outcomes, with crocin and vaspin treatment dramatically lowering fasting insulin levels.

In addition, we estimated the HOMA-IR, which reflects insulin resistance, which is common in T2DM, obesity, and metabolic syndrome. Homeostatic Model Assessment of insulin resistance is a method that uses baseline (fasting) glucose and insulin or C-peptide concentrations to determine IR. Generally, HOMA-IR less than one reflects excellent insulin sensitivity. In adults, a HOMA-IR value of 2.5 is considered indicative of IR.^37^ The present study revealed a significantly higher HOMA-IR value in the untreated HFD-STZ diabetic rats compared to normal rats. After intervention with GLE for 14 consecutive days, animals given a greater dose of GLE showed a substantial reduction in HOMA-IR compared to untreated control rats.

The role of oxidative stress in insulin resistance has only lately been established. Excess endogenous reactive species cause oxidative stress by damaging cells and disrupting signal pathways.^38^ Reactive species, in particular reactive oxygen species (ROS) such as hydrogen peroxide, hydroxyl radical ions, andsuperoxide ions, which are generated at small physiological concentrations primarily in mitochondria and peroxisomes, are the agents of oxidative stress.^39^ Antioxidants can control autoxidation by halting the propagation of free radicals or limiting the free radicals generation, reducing oxidative stress, improving immunological function, and extending healthy longevity. Hyperglycemia causes the formation of free radicals and compromises the endogenous antioxidant defence system in diabetic individuals. In the body, there are antioxidant defense systems that are both enzymatic and non-enzymatic in nature. Their role in human cells is to counteract the harmful effects of ROS. Antioxidants include non-enzymatic like ascorbic acid, retinol, vitamin E, and GSH, as well as the enzymes such as SOD, CAT, GPx, and GRx.^40^ In the present study, GLE intervention resulted in the mitigation of pancreatic oxidative damage by increasing endogenous antioxidant levels (SOD, CAT, and GSH), which was parallel with the results of standard metformin. These effects could be attributed to its antihyperglycemic effect, decreased insulin resistance, and improved glycemic control. Similar findings have been previously reported.^36^

### Study limitations

Short-term GLE therapy for 2 weeks is one of the study’s limitations. The treatment course is suggested to be extended to identify the possible chronic effects of GLE in T2DM rats. The problem of extrapolating pharmacological data from preclinical to clinical must not be neglected.

In conclusions, GLE intervention enhanced glycemic control, body weight change, serum insulin levels, glucose tolerance, HOMA-IR, and endogenous antioxidant levels in HFD-STZ T2DM rats. Our findings suggest that *G. latifolia* is the ideal medicinal plant candidate for treating T2DM and that it should be investigated further. However, more molecular research is required to comprehend its probable mechanism fully.
